# Epileptic Seizure Detection Based on Variational Mode Decomposition and Deep Forest Using EEG Signals

**DOI:** 10.3390/brainsci12101275

**Published:** 2022-09-22

**Authors:** Xiang Liu, Juan Wang, Junliang Shang, Jinxing Liu, Lingyun Dai, Shasha Yuan

**Affiliations:** School of Computer Science, Qufu Normal University, Rizhao 276826, China

**Keywords:** electroencephalography, seizure detection, variational modal decomposition, log−Euclidean covariance matrix, deep forest

## Abstract

Electroencephalography (EEG) records the electrical activity of the brain, which is an important tool for the automatic detection of epileptic seizures. It is certainly a very heavy burden to only recognize EEG epilepsy manually, so the method of computer-assisted treatment is of great importance. This paper presents a seizure detection algorithm based on variational modal decomposition (VMD) and a deep forest (DF) model. Variational modal decomposition is performed on EEG recordings, and the first three variational modal functions (VMFs) are selected to construct the time–frequency distribution of the EEG signals. Then, the log−Euclidean covariance matrix (LECM) is computed to represent the EEG properties and form EEG features. The deep forest model is applied to complete the EEG signal classification, which is a non-neural network deep model with a cascade structure that performs feature learning through the forest. In addition, to improve the classification accuracy, postprocessing techniques are performed to generate the discriminant results by moving average filtering and adaptive collar expansion. The algorithm was evaluated on the Bonn EEG dataset and the Freiburg long−term EEG dataset, and the former achieved a sensitivity and specificity of 99.32% and 99.31%, respectively. The mean sensitivity and specificity of this method for the 21 patients in the Freiburg dataset were 95.2% and 98.56%, respectively, with a false detection rate of 0.36/h. These results demonstrate the superior performance advantage of our algorithm and indicate its great research potential in epilepsy detection.

## 1. Introduction

Epilepsy is a chronic disease of the brain, which is mainly caused by the disturbance of neurons in the brain [1]. Over the past few decades, more than 50 million patients worldwide have been severely disrupted by epilepsy [2]. In 2017, the International League Against Epilepsy (ILAE) classified seizure types into focal epilepsy, generalized epilepsy, and epilepsy of unknown symptoms [3]. With the development of medicine, various antiepileptic drugs are of great help for the treatment of epilepsy, but there are still 25–50% of epilepsy patients with persistent seizures [4]. EEG can accurately describe the fluctuation of information in the brain, which records a large amount of pathological information about epileptic seizures and is very helpful in the diagnosis and treatment of epilepsy [5,6]. However, the number of EEGs is very large, and relying solely on visual inspection by an experienced medical expert will bring a huge burden. Therefore, automatic epilepsy detection technology has a profound impact on reducing the pressure on medical personnel and promoting the progress of epilepsy treatment.

The traditional automatic seizure detection technology mainly consists of feature extraction and a classifier [7,8,9]. Usually, feature extraction is an indispensable and important step in seizure detection. Sharma et al. [10] accomplished the job of seizure classification based on the iterative filtering (IF) of EEG signals and extracted the amplitude envelope (AE) function as EEG features. You et al. [11] combined a flexible analysis wavelet transform (FAWT) with logarithmic energy entropy (LEE) and fuzzy distribution entropy (fDistEn) to realize the classification of EEG signals. Follis et al. [12] used an autoregressive moving average−generalized autoregressive conditional heteroscedasticity (ARMA−GARCH) model to collect the volatility half-lives of different EEG channels for a better understanding of seizure processes.

It is well-known that time–frequency analysis is the cornerstone of seizure detection systems [13,14]. It can capture the transient information of nonstationary signals in the time and frequency domains, paving the way for subsequent feature extraction. Phadikar et al. [15] combined wavelet packet decomposition (WPD) and improved nonlocal mean (NLM) algorithm to denoise EEG signals. Ahmed et al. [16] used short−time Fourier transform (STFT) to obtain the frequency spectrum of EEG signals and extracted the differential entropy as an EEG feature. Variational mode decomposition (VMD) is an adaptive time–frequency analysis method that could solve the nonstationary characteristics of EEG signals [17]. This method has the advantage that the number of modal decompositions can be adaptively determined according to the existing form of the signal. During the variational solution process, the method adaptively surrounds the center frequency of the mode to achieve an effective separation of the variational modal functions (VMFs). Rout et al. decomposed the EEG signal into a series of VMFs through VMD, extracted five instantaneous features with the help of the Hilbert transform (HF), and put the features into the error-minimizing random vector functional link network (EMRVFLN) to complete the EEG classification of the signals [18].

The covariance matrix can characterize the correlation between features and is currently used in many fields to study time series [19]. Abdulla et al. designed a covariance matrix decision model that combines Kolmogorov–Smirnov and Mann–Whitney U tests to rank EEG features and used the AdaBoost backpropagation neural network to achieve the classification of EEG signals [20]. Lei et al. applied symmetric positive definite (SPD) matrices in the form of covariance descriptors to present EEG signals and proposed the discriminative Stein kernel−based sparse representation for seizure detection [21].

The development of deep learning has led to advances in areas such as image recognition, EEG analysis, and seizure detection [22,23,24]. Truong et al. put the time–frequency information obtained by the short-time Fourier transform (STFT) into CNN to classify the nonseizure and seizure periods [25]. Singh et al. used a modified grasshopper optimization algorithm (GOA) and an artificial neural network (ANN) to identify EEG signals [26]. Deep learning can automatically learn the intrinsic characteristics of EEG signals from an end−to−end perspective to complete the classification of EEG signals. Deep forest (DF) is a new deep ensemble method proposed by Zhou et al., which combines deep neural networks and decision trees [27]. The DF model includes two parts, namely multigranularity scanning and cascade forest. Cascading forests could realize the layer-by-layer analysis of features, and multigranularity scanning is beneficial to enhance the connection between data features. Compared with the deep neural network, the parameter adjustment of tree-based DF is easier, and it also has good performance in different fields under the same parameters [28,29]. Recently, Cao et al. classified hyperspectral images (HSI) with the help of rotation-based deep forest (RBDF) [30]. It can be seen from the experimental results of three HSIs that RBDF can enhance the recognition ability of spectral features and improve classification accuracy. Fang et al. used a combination of power spectral density (PSD) and differential entropy (DE) as the input of a deep forest to identify emotions in EEG signals [31].

The purpose of this work is to study an innovative automatic seizure detection algorithm. EEG time−frequency information using variational modal decomposition is helpful for epilepsy detection. Through LECM, the Riemannian manifold of the covariance matrix could be mapped to linear space, and the difference between nonseizure and seizure signals could be distinguished. The deep forest classifier is used for more effective recognition. Finally, we evaluate the performance of this algorithm on two EEG datasets. The rest of the paper is structured as follows: The two EEG databases are introduced in Section 2, and Section 3 describes the specific implementation of this algorithm, including preprocessing, deep forest, and postprocessing. The evaluation criteria and experimental results are presented in Section 4. A discussion of the results is provided in Section 5, followed by the conclusions in Section 6.

## 2. EEG Datasets

### 2.1. Bonn Dataset

The Bonn dataset contains five sets: Z, O, N, F, and S, and each set consists of 100 time series that were sampled at 173.6 Hz and are 23.6 s in duration [32]. The S, F, and N sets were collected using electrodes placed intracranially, where F and N were the nonseizure signals placed in the epileptic area and contralateral hippocampus, and S was the seizure signal for five epilepsy patients. The Z and O sets were collected using scalp electrodes, which contained the EEG signals of five healthy volunteers with their eyes open and closed, respectively. In this paper, the two sets S and F were applied to the binary classification problem of intracranial EEG signals.

### 2.2. Freiburg Dataset

The Freiburg EEG dataset came from the Epilepsy Center at the University Hospital Freiburg, Germany, and contains intracranial EEG recordings from 21 patients with strong resistant focal epilepsy. The sampling frequency of this dataset is 256 Hz, and it was collected by a Neurofile NT digital video monitor. In this dataset, experienced experts marked the beginning and end of seizures in each patient, and the EEG information of each patient was collected from six channels. Three channels were placed in the focal area, and three channels are placed in the extrafocal area. In this work, we only studied the three channels located in the focal region.

Due to differences in the types of seizures and the factors that cause seizures, the duration of the seizure activity is different for patients. Long seizure activity can last up to 15 min, while short seizure activity lasts only 12 s. Experts use the clinical presentation of epilepsy patients to mark when each seizure event begins and ends. In total, this dataset contains 87 seizure events, and the mean seizure duration is 114.3 s. More detailed data about the Freiburg dataset can be found in reference [33]. In our experiments, one or more seizure events and same amount of nonseizure EEG data were randomly selected for each patient for training, and the rest of the EEG data were used to test the trained model. A total of 83.35 min of seizure data (including 33 seizure events) and 3.6 h of nonseizure data were used for 21 patients as a training dataset. In the testing set, there were 116.7 min of epileptic seizure data (including 54 seizure events) and 648.57 h of nonseizure data.

## 3. Methods

**Figure 1:**
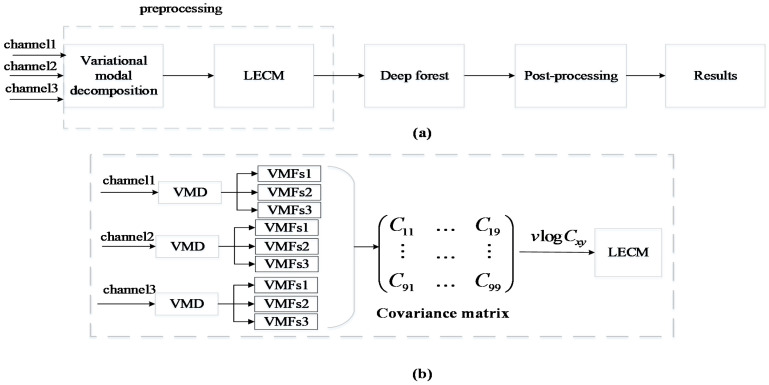
Block diagram of this automatic seizure detection algorithm. (**a**) The complete flow of the whole system, including preprocessing, classification, and postprocessing. (**b**) The process of preprocessing in detail, including VMD and the calculation of LECM, where represents the logarithmic half-vectorization operation.

The framework of our proposed epilepsy seizure detection algorithm is shown in Figure 1, which mainly consists of three parts: preprocessing, deep forest classification, and postprocessing. We describe each procedure in detail in the following subsections.

### 3.1. Preprocessing

Since the EEG signal is dynamic and nonstationary, the EEG signal of each channel is divided into 4 s epochs with a moving window without overlap [34].This window could capture epilepsy activity well and differentiate EEG signals while maintaining the stationarity of the signals. In our work, preprocessing mainly includes two processes. The first step is to apply variational modal decomposition (VMD) to find the predominant low-frequency region of seizures. Then, the log−Euclidean covariance matrix (LECM) is designed and applied to reduce the dimension of the EEG signal and further highlight the characteristics of the EEG signal.

#### 3.1.1. Variational Modal Decomposition

Due to the good robustness and adaptability, variational modal decomposition (VMD) can better analyze and handle nonlinear and nonstationary signals such as EEG, which adaptively decompose the original signal, *f*(*t*), into non-recursive modes $u_{k},k=1,2,\ldots ,K$. Each mode is called a variational mode function (VMF) and can be expressed as Equation (1) [35]:

(1) $$u_{k}=A_{k}\cos(\phi_{k})$$

Here, the envelope of the VMFs is *A_k_*, and $\phi_{k}$ is the phase. The derivative of the envelope of the VMFs is the central frequency $\omega_{k}$, $\omega_{k}={\phi \prime }_{k}$, and each VMF is centered on the central frequency.

When VMD deals with variational problems, it first analyzes each mode, *u_k_*, through the Hilbert transform to obtain the unilateral spectrum $\left(\delta (t)+j/\pi t)*u_{k}(t\right)$ and then adds $e^{-j\omega_{k}t}$ for each mode, $u_{k}$, to adjust the center frequency, *ω_k_*. Finally, through the action of Gaussian smoothing, we obtain a constrained variational problem, as shown in Equation (2) [36]:

(2) $$\begin{array}{cc} \min_{\left\{u_{k}\},\{\omega_{k}\right\}}\{\sum_{k}^{}\|\partial_{t} & [(\delta (t)+j/\pi t)*u_{k}(t)]e^{-j\omega_{k}t}{\|}_{2}^{2}\}\\ & s.t.\sum_{k=1}^{K}u_{k}=f(t) \end{array}$$

where $\partial_{t}$ denotes the partial derivative, is the unit impulse function, and f(t) is the original signal, which is the summation over all modes. Combined with the data fidelity constraint factor, *α*, and the Lagrangian multiplier, λ(t), to form an augmented Lagrangia, (L), the constrained variational problem is transformed into an unconstrained variational problem [37].

(3) $$\begin{array}{l} L(\{u_{k}\},\{\omega_{k}\},\lambda )=\alpha \sum_{k}^{}{\left\|\partial_{t}[(\delta (t)+j/\pi t)*u_{k}(t)]e^{-j\omega_{k}t}\right\|}_{2}^{2}+\\ {\left\|f(t)-\sum_{k}u_{k}(t)\right\|}_{2}^{2}+\langle \lambda (t),f(t)-\sum_{k}^{}u_{k}^{}(t)\rangle \end{array}$$

The sequence is updated using the alternative direction method of multipliers (ADMMs) algorithm to solve the variational problem in the decomposition process [38], where the mode, $u_{k}$, and the center frequency, $\omega_{k}$, are updated as [39]:

(4) $${\hat{u}}_{k}^{n+1}(\omega )=\frac{\hat{f}(\omega )-\sum {}_{i\neq k}{\hat{u}}_{{}_{i}}(\omega )+\hat{\lambda }(\omega )/2}{1+2\alpha {\left(\omega -\omega_{k}\right)}^{2}}$$

(5) $$\omega_{k}^{n+1}=\frac{{\int_{0}^{\infty }\omega \left|{\hat{u}}_{k}^{n+1}(\omega )\right|}^{2}d\omega }{{\int_{0}^{\infty }\left|{\hat{u}}_{k}^{n+1}(\omega )\right|}^{2}d\omega }$$

In this way, the original signal, f(t), is decomposed into a series of modal functions, $u_{k}$, containing the time–frequency information of the EEG signal. Reference [40] provides a complete variational modal optimization scheme.

Compared with other time–frequency analysis methods, VMD has good robustness and adaptability, which can perfectly analyze nonlinear and nonstationary EEG signals. VMD always decomposes low−frequency information first and then decomposes high−frequency information, which is helpful for seizure detection since the characteristics of epileptic seizures are more prominent in the low-frequency part of the EEG signal [41]. The VMFs decomposed by VMD are closely related to the center frequency, so the center frequency of the VMFs would gradually increase. Table 1 and Table 2 show the distribution of center frequencies with different modal numbers for the Bonn and Freiburg datasets, respectively. It can be seen that when K = 6 the center frequency does not continuously increase but first increases and then decreases, indicating that there is excessive decomposition. Therefore, we chose the modal number K = 5 for these two datasets.

**Figure 2:**
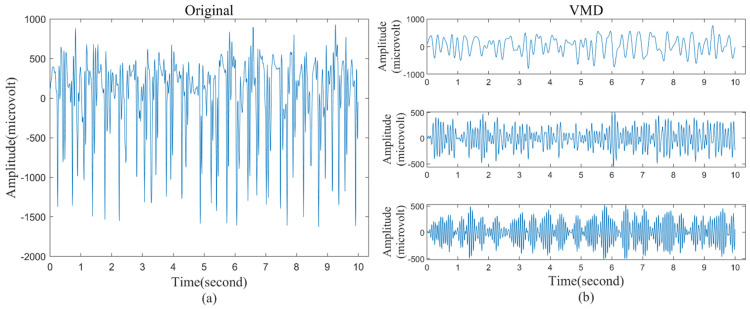
A piece of the original signal during the seizure period and the subsignals decomposed using VMD. (**a**) Original signals; (**b**) subsignals decomposed by VMD (VMF1, VMF2, and VMF3).

An increase in the number of VMD decomposition layers leads to the appearance of false VMFs. In addition, the low-frequency portion of the EEG signal often contains more useful information for seizure detection [42]. To reduce the interference of spurious VMFs components, we analyzed and calculated the correlation coefficient between each VMF and the original signal, as shown in Table 3. In both datasets, the correlation coefficients of VMF4 and VMF5 were not greater than 0.3, indicating that they contained less original information. To improve the classification accuracy, we only kept VMF1, VMF2, and VMF3 for subsequent EEG signal classification. Figure 2 is the comparison between the original signal and VMF1, VMF2, and VMF3 when K = 5 in the Bonn dataset.

#### 3.1.2. Log-Euclidean Covariance Matrix

The covariance matrix is an important descriptor for EEG signals, which could reflect the second-order statistical properties of elements and describe multiple types of information and correlations of features. The covariance matrix is usually a symmetric positive definite (SPD) matrix, while the space of the SPD matrix is not a linear Euclidean space but a nonlinear Riemannian manifold. Therefore, an SPD matrix cannot perform Euclidean operations directly and can be operated in two ways, the affine-invariant Riemannian framework and the log-Euclidean Riemannian framework [43,44,45]. Both frameworks have good theoretical foundations, but the computational cost of the former is higher than that of the latter. The following mainly describes the calculation of LECM using the log−Euclidean framework.

The space of a symmetric positive definite has the characteristics of the Riemannian manifold, which can be mapped to Euclidean space through logarithmic operations. Li et al. showed that logarithmic SPD matrices can be processed by simple Euclidean operations [46]. This greatly facilitates the statistical analysis of the SPD matrix. Each SPD matrix satisfies the decomposition of Equation (6) [47].

(6) $$C=U\Lambda U^{T}$$

Based on the log−Euclidean framework, for any SPD matrix, C**,** there is a unique corresponding logarithmic operation, logC [46]:

(7) $$\log C=U\cdot Diag(\log(\lambda_{1}),\ldots ,\log(\lambda_{n}))\cdot U^{T}$$

Here, U is an orthogonal matrix and $\Lambda =Diag(\lambda_{1,}\ldots ,\lambda_{n})$ is a diagonal matrix including the eigenvalues $\lambda_{i},i=1,\ldots ,n$ for C. After calculating Equation (7), we can obtain this log−Euclidean covariance matrix (LECM).

According to the symmetric characteristics of LECM, we perform a semivector operation on log*C*, where we only keep its diagonal elements and the elements above it, with the elements below the diagonal deleted. Thus, a vector, $v\log C_{xy}$, is formed by the column as extracted EEG features, which can be expressed as:

(8) $$v\log C_{xy}=[\log C_{11},\log C_{12},\log C_{22},\ldots ,\log C_{nn}]$$

**Figure 3:**
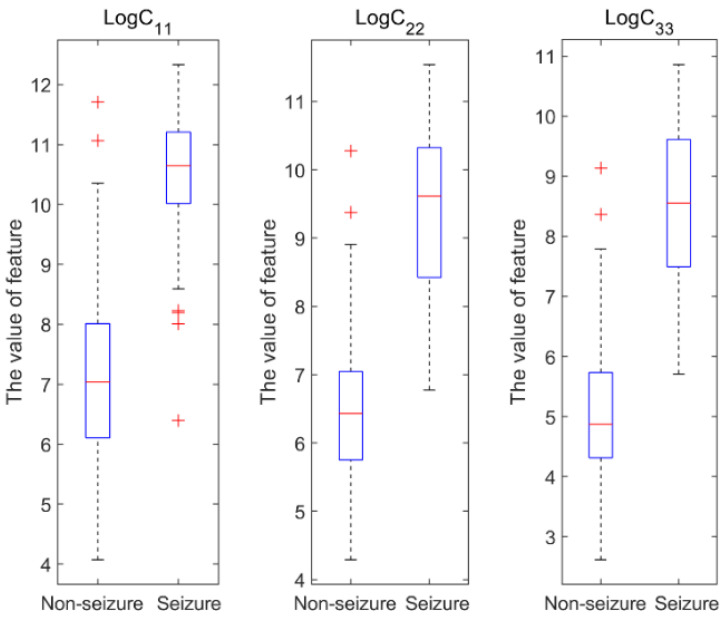
Boxplots of the diagonal elements of the LECM of the Bonn dataset.

In this work, the variational modal functions (VMFs) of the VMD decomposition were used to construct the EEG time–frequency distribution matrix and calculate its covariance matrix, *C*. Then, we extracted a vectorized LECM to characterize the EEG signals. As illustrated in Figure 3, the boxplots of the diagonal elements of the LECM were analyzed to compare the distributions of seizure and nonseizure EEG data in the Bonn dataset, which shows that the two types of EEG data are significantly different.

### 3.2. Deep Forest

**Figure 4:**
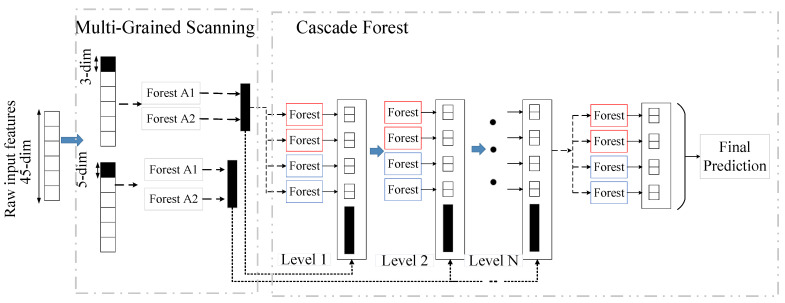
The complete frame process of the deep forest structure. Each layer of the cascade forest consists of two random forests (red) and two full random forests (blue).

Deep forest is an extension and deep model of random forest, which combines the idea of deep learning based on random forest. Deep forests learn how deep neural networks handle feature relationships, but unlike deep neural networks, deep forests do not have many hyperparameters, and deep forests also perform well on small sample datasets [48]. Figure 4 depicts the overall framework of deep forest. The sliding window of multigranularity scanning is used to extract the original features and enhance the correlation between the features. The sequences obtained by scanning processing are used as the input of Forest A1 and Forest A2, and the corresponding class probability of each input is obtained by the forest. The results of the forest output are concatenated as the input of the cascade forest.

Cascade forests are an ensemble of decision trees. To enhance the generalization ability of cascade forest, each layer consists of two different forests, full random forest and random forest. The level in the cascade forest is adaptive and does not need to be manually set in advance. This is because it performs performance evaluation at the end of one level and then trains at the next level; when the training is over, the entire cascade is evaluated again. If there is no significant improvement in performance, the training process terminates. The adaptability of the cascade forest can determine the complexity of its model, which makes the deep forest have outstanding performance in the face of small datasets, such as EEG data, compared to DNN.

In the procedure of multigranularity scanning, the choice of window size needs to consider the dimension of the input features. The window should not be too small, as the amount of data after scanning would be too large and cause computational burden, and the window should not be too large, as effective feature correlation information cannot be captured. After the LECM, the original EEG feature vector of the Bonn dataset was 6−dimensional and the original feature vector of the Freiburg dataset was 45−dimensional. Considering that the window length cannot exceed the original feature dimension, the length of one larger window was set to 5 and the other smaller window was set to 3 for both datasets.

**Figure 5:**
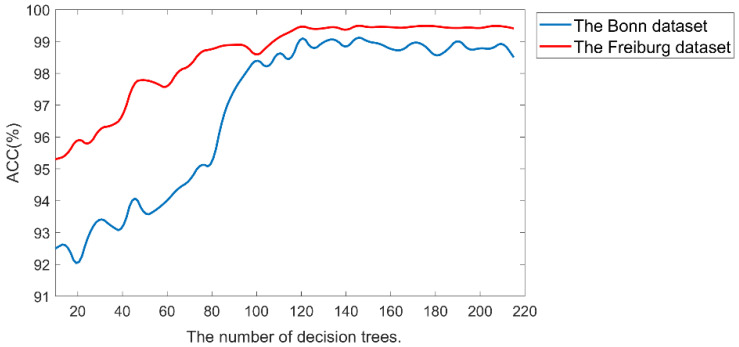
The effect of the number of decision trees on the classification results of the deep forest model for two datasets.

The deep forest is a model based on RF and combined with the layer structure of deep learning. We all know that decision trees are the core of RF, and the number of trees is an important parameter in RF, which affects the performance of classification. Too many trees can increase the computational burden. To clarify the appropriate number of decision trees in the deep forest model, we analyzed the results of the EEG classification by performing 10−fold cross−validation experiments with different numbers of trees in the Bonn and Freiburg datasets. Figure 5 provides the relationship between the number of decision trees and the classification accuracy for the two datasets. Among them, the Freiburg EEG database is a long-term EEG dataset, and the seizure duration of each patient is very short, much smaller than the nonseizure EEG data. Therefore, 10−fold cross−validation is not well-suited for the entire EEG dataset. Therefore, we randomly chose 10 patients from the 21 patients and performed a cross-validation experiment combining all seizure data of these patients with randomly selected nonseizure data of the same length. As can be seen in the Figure 5, at first the classification accuracy was effectively improved as the number of trees increased. When the number of trees was below the range of 100, the accuracy improved very rapidly, but when the number of trees increased to 120, the classification accuracy stabilized and did not change significantly. Hence, we set the number of trees to 120 to save computational costs while ensuring good classification performance.

### 3.3. Postprocessing

To improve the classification accuracy, we utilize postprocessing techniques to process the output of the deep forest, which mainly include a probabilistic output subtraction operation, smoothing, threshold judgment, and adaptive collar technology. The judgments of the deep forest model on the nonseizure period and the seizure period of the EEG signal are two probabilistic outputs. To effectively distinguish two different types of EEG signals, the final probabilistic result is the difference obtained by subtracting the two output values.

The linear moving average filter (MAF) is used to remove sudden glitches in the probabilistic output of the deep forest [49]. Although this filter is simple, it has good performance, which not only effectively removes noise, but also retains the sharpest part to a certain extent. It is helpful for our seizure detection to reduce some short-lived wrong decisions, which can be defined as:

(9) $$y_{k}=\frac{1}{2M+1}\sum_{i=-M}^{M}{\hat{y}}_{k+i}$$

where $\hat{y}$ is the probabilistic result, *y* is the filtered signal, and 2M+1 represents the smoothing length. Then, comparing the threshold with the smoothed output yields a binary decision: 0—nonseizure or 1—seizure. Since the average seizure duration of each patient is different, the smooth length is also different and is determined by obtaining the best classification result of the training data. In the training stage, we set different smoothing lengths successively with a limitation to a maximum of 35 to find the optimal parameters, and the length was fixed in the testing stage for this patient. In addition, the threshold for each patient was also determined during the training phase using a similar method.

**Figure 6:**
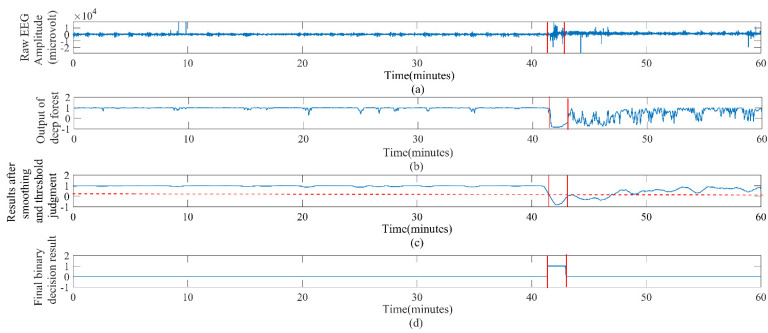
Example of successful seizure event detection with completed postprocessing technique for patient 20. (**a**) One hour of raw EEG signals. (**b**) The output of the deep forest classifier. (**c**) The results after the smoothing operation and the threshold judgment. The horizontal red dotted line is the threshold. (**d**) The final binary classification results after the adaptive collar technology, where the period between the two red vertical lines is the epileptic seizure period marked by the expert.

In addition, the evolution of epileptic seizure is a continuous dynamic process. The characteristics of the beginning and end phases of seizures are not much different, and the two phases are made less clear by the smoothing operation. In response to this problem, adaptive collar technology can reduce the missed part of the seizure [50]. Figure 6 presents an example of an epileptic event successfully detected in patient 20 with a complete postprocessing technique.

## 4. Results

In this part, we mainly introduce our experimental evaluation criteria and results. This experiment was implemented in MATLAB 2018 and the Python 3.7 environment. First, in the preprocessing stage, we used VMD to perform a time–frequency analysis of EEG signals, determined the number of modes used by calculating the cross-correlation coefficient between each modal component and the original signal, and constructed the time–frequency distribution matrix of EEG signals. Then, the LECM was calculated, the corresponding EEG features were extracted, and we put them into the proposed deep forest model to obtain the probability output. Finally, the postprocessing techniques, such as smoothing and the adaptive collar techniques, were performed on the probabilistic outputs to improve the classification accuracy.

### 4.1. Evaluation Criteria

This paper mainly adopts two evaluation criteria, which are epoch-based criteria and event-based criteria [51]. The epoch-based evaluation criteria include four indicators: sensitivity, specificity, accuracy, and G-mean, which are represented as follows:

(10) $$Sensitivity=\frac{TP}{TP+FN}$$

(11) $$Specificity=\frac{TN}{TN+FP}$$

(12) $$Accuracy=\frac{TP+TN}{TP+FP+TN+FN}$$

(13) $$G-mean=\sqrt{Sensitivity*Specificity}$$

Among them, *TP* represents the number of seizures correctly detected by the algorithm, *FN* is the number of seizures incorrectly determined by the algorithm to be nonseizures, *TN* indicates the number of nonseizures correctly detected by the algorithm, and *FP* is the number of nonseizures incorrectly determined by the algorithm to be seizures. Thus, sensitivity is applied to evaluate the ability of our algorithm to classify seizure *EEG* data, while specificity can evaluate the ability to classify nonseizure *EEG* data. For the imbalance in seizure and nonseizure data in the Freiburg dataset, *G*−*mean* can be a good evaluation of the algorithm’s classification effect on two different types of data.

The event-based sensitivity and false detection rate constitute the event-based evaluation criteria. Event-based sensitivity is obtained by calculating the number of true seizure events detected and then dividing by the total number of expert−labeled seizure events. The system judges one or more consecutive EEG signals in the nonseizure period as the signal of the nonseizure period, which is called a false positive, and the false detection rate is the average number of false positives per hour.

### 4.2. Experiment Result

**Figure 7:**
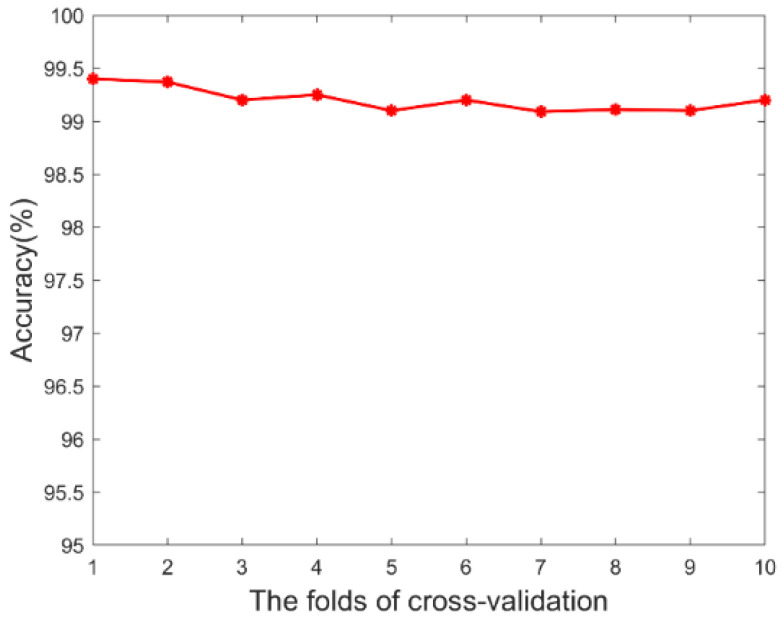
The performance obtained by 10-fold cross-validation on the Bonn dataset.

In the Bonn dataset, the 10-fold cross-validation experiments were applied to evaluate the algorithm performance, which could avoid overfitting to a certain extent. The two classes of intracranial EEG signals were divided into 10 nonoverlapping subsets of the same size. Then, 9 subsets were used as the training dataset, 1 subset was used as the testing dataset, and 10 experiments were carried out in turn. Finally, the average of 10 results was calculated. Figure 7 shows the performance obtained by the 10−fold cross−validation on the Bonn dataset. The average of sensitivity, accuracy, and specificity were 99.2%, 99.31%, and 99.26%, respectively, indicating that our algorithm has good classification performance between the seizure and nonseizure periods.

**Figure 8:**
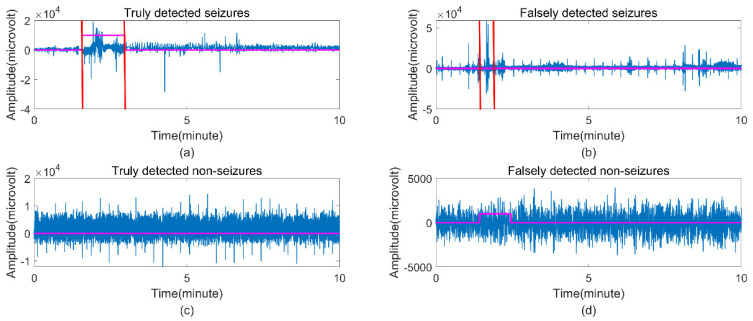
Four EEG time series with different judgment results of this seizure detection system. (**a**) Truly detected seizures; (**b**) falsely detected seizures; (**c**) truly detected nonseizures; (**d**) falsely detected nonseizures. The seizures identified by the experts are represented between the vertical red lines, and the purple horizontal line represents the binary decision result of our algorithm.

Table 4 and Table 5 show the epoch-based and event-based performance on the Freiburg dataset, respectively. Table 4 represents the average values of epoch-based sensitivity, specificity, accuracy, and G-mean of 95.2%, 98.56%, 98.52%, and 96.64%, respectively. Most of these patients (patients 1, 2, 3, 5, 6, 7, 11, 12, 13, 15, 16, 17, and 20) had a maximum sensitivity of 100%. Only patients 18 and 19 had sensitivity values lower than 80%, which were not satisfactory results. The specificity of 10 patients was above 99%, and the specificity of all 20 patients, except patient 10, was higher than 96%. There were 11 patients (patients 1, 5, 6, 7, 11, 12, 13, 15, 16, 17, and 20) whose G-mean index exceeded 99%. Only patients 4, 10, 18, and 19, with less than 96%, did not achieve our expected results. Event-based evaluation criteria tend to be more in line with clinical practice. We used 54 seizure events as our test set to evaluate the ability of the epilepsy detection system. There were 51 events detected, three of which were missed, with an event−based average sensitivity of 94.44%. Figure 8 shows the four EEG time series with different judgment results of the seizure detection system to which our proposed method was applied.

From Table 5, we can also observe that the average false detection rate of the event-based evaluation metric, which also performed well, was 0.36/h. Only patient 18 had a false detection rate greater than 1/h because the seizure duration of patient 18 was too short. It was less than 15 s, so the system could not extract accurate features. Large-scale rhythmic epileptic activity is the main cause of most false detections, and nonseizure signals are judged as seizure signals.

## 5. Discussion

**Figure 9:**
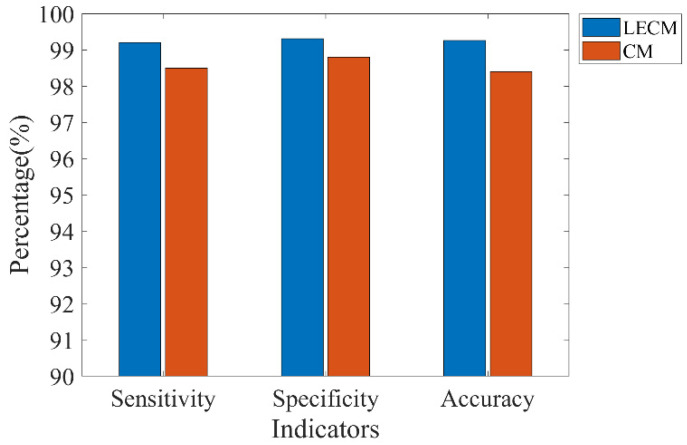
Classification results of LECM and CM using deep forest in the Bonn dataset.

An effective seizure detection system must have both high sensitivity and high specificity as well as a low false detection rate. The covariance matrix (CM) is an important tool for EEG signal analysis, which can effectively distinguish different types of EEG signals. In this paper, this ability of the covariance matrix is greatly enhanced by computing the LECM, which indirectly maps the covariance matrix to the Euclidean space. We compared the direct use of the covariance matrix of the EEG features with the proposed LECM features on the Bonn dataset. Figure 9 shows the classification results of these two feature vectors under deep forest using the same parameters. It can be seen that, compared with directly using the covariance matrix as the EEG features, the LECM has better ability in seizure detection.

Deep forest is a tree-based deep neural network model. To verify its performance, we used support vector machine (SVM), random forest (RF), Bayesian linear discriminant analysis (BLDA), and K−nearest neighbor (KNN) to compare with the deep forest model under the same input. Among them, BLDA adopts a regularization method to avoid overfitting in high-dimensional data and noisy data and does not need manual parameter tuning. We only set the average value of the BLDA output as the final classification threshold. RF builds multiple decision trees and uses them to vote for classification results. The number of decision trees in the RF is the same as the number selected in the deep forest model. The core idea of SVM is to find the best hyperplane between samples with different attributes to classify the data. Here, we used the Gaussian function and applied the transformation technique to determine the hyperparameters. During the testing process of KNN, the method determines the class of the sample based on the class of one or several recent samples. In this experiment, the number, K, of adjacent classes was taken to be 5.

**Figure 10:**
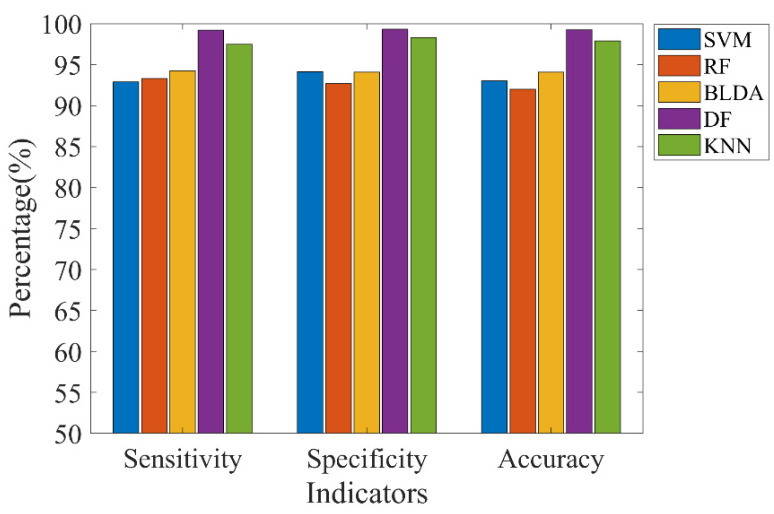
Classification results of five different classifiers using LECM features on Bonn dataset.

As shown in Figure 10, the sensitivity, specificity, and accuracy obtained by the deep forest classification were all higher than the other four classifiers, which shows that deep forest has more ability in the classification of EEG signals than the other classifiers and is more sensitive for the recognition of EEG signals. Certainly, the ability of deep forest to achieve such excellent performance is the result of the joint action of the LECM and this deep forest model. Therefore, we will further explore more features of deep forest to verify the performance of the model in future work.

The Freiburg dataset used in this paper has been used to validate the effectiveness of epilepsy detection algorithms by many researchers. Table 6 presents a comparison of the results of our work with other studies using the Freiburg EEG dataset. Yan et al. introduced an epileptic seizure detection method based on the Stockwell transform combined with power spectral density and using a gradient boosting algorithm as a classifier [52]. The epoch-based sensitivity, specificity, and accuracy of their system reached 94.26%, 96.34%, and 98.30%, respectively, and the event-based false detection rate was 0.66/h, all lower than our algorithm. In addition, they only studied 20 of these patients, and did not analyze patient 10.

Recently, Mahmoodian et al. combined cross-bispectral features and support vector machines to achieve automatic epilepsy detection [53]. Although their false detection rate of 0.24/h was lower than our algorithm, they only trained on 20 patients and ignored patient 13. The epoch−based sensitivity and specificity were 95.83% and 98.56%, respectively. Tzimourta et al. realized the recognition of epileptic seizures based on wavelet transform and support vector machine [54]. While their classification works well, they only tested 21 patients for a total of 28.6 h, which is lower than our algorithm’s performance of 650.52 h on 21 patients. Mu et al. used wavelet transform to obtain the time–frequency distribution matrix of EEG signals and combined graph-regularized non-negative matrix factorization to distinguish the seizure and nonseizure signals [55]. Although our algorithm is lower in specificity than theirs, we have high sensitivity and a lower false positive rate than their algorithm. Based on the end-to-end idea of deep learning, Hussain et al. proposed a deep learning hybrid structure that is a combination of CNN and LSTM [56]. In their work, they did not perform feature extraction but directly put the temporal information of the original EEG signal into a hybrid model of CNN and LSTM to classify the EEG signal. The results they obtained had averages of 94.71%, 93.99%, and 90.53% for sensitivity, specificity, and accuracy, respectively.

In the study of Abugabah et al., the first 10 patients in the Freiburg dataset were analyzed, 15 statistical features were screened with the help of the krill swarm algorithm, and the distinction between seizures and non-onsets was completed under the artificial algae optimization neural network, which finally obtained 98.9% accuracy [57]. Malekzadeh et al. proposed a seizure detection algorithm that combines handcrafted and deep learning features [58]. In their work, tunable−Q wavelet transform (TQWT) is used to obtain subband EEG information, and various linear and nonlinear features are calculated. Then, a deep learning method of CNN−RNN was proposed to complete seizure detection, which achieved very good results, with an accuracy rate of 99.13%. Although its accuracy exceeds that of our method, the use of training and testing data in their study were not illustrated. Moreover, our algorithm is much simpler than their work and achieves remarkable results on a very small training set. As can be seen in Table 6, we propose a more prominent epilepsy detection method for distinguishing seizure and nonseizure periods, and our method was used in the Bonn and Freiburg datasets, indicating that our algorithm has better generalization ability.

Furthermore, with the development of long-term EEG monitoring technology, more and more EEG recordings need to be analyzed, and the imbalance between seizure and nonseizure data needs to be further considered in this proposed deep forest model. In addition, some factors such as the location of the electrodes, the age of the patient, and the type of epilepsy can affect the detection results. Although this experiment did not distinguish these, indicating that our algorithm has good adaptability, the influence of these factors still needs to be explored. Moreover, our experimental results still need to take into account the limitations of the EEG datasets used, such as scalp EEG recordings. Although our proposed method is very effective, there are definitely some differences and limitations for clinical practice, so we need to further improve and validate our algorithm on larger EEG datasets to make it suitable for clinical use.

## 6. Conclusions

This paper introduces an automatic seizure detection algorithm based on variational modal decomposition and deep forest models. The adaptability of VMD can not only obtain time–frequency information that is useful for epilepsy detection, but it also effectively distinguishes two different EEG signals during the seizure and nonseizure phases. Compared with directly using the covariance matrix as the eigenvector, the LECM not only preserves the spatial structure of the covariance matrix but also avoids the high computational burden of the Riemannian manifold through indirect mapping. The detection capability of the LECM has a more obvious advantage. The experimental results show that the classification based on the deep forest model is very effective, with a low misjudgment rate. Therefore, our method is very effective in the detection of intracranial EEG and has great development prospects in other biological information fields. Considering the adaptability of this algorithm and the limitations of the EEG data, in future work we will further focus on improving the deep forest model and integrating more available features to improve the performance of the seizure detector for clinical application.

## Figures and Tables

**Table 1:** The central frequencies corresponding to different modal function numbers in the Bonn dataset.

Number of Modes	Center Frequency (Hz)					
2	0.47	10.40	−	−	−	−
3	0.32	6.60	22.82	−	−	−
4	0.30	5.97	17.88	68.89	−	−
5	0.26	5.45	14.46	29.22	72.51	−
6	0.26	5.32	13.63	40.17	74.60	24.86

**Table 2:** The central frequencies corresponding to different modal function numbers in the Freiburg dataset.

Number of Modes	Center Frequency (Hz)					
2	1.08	16.51	−	−	−	−
3	0.54	11.44	34.53	−	−	−
4	0.74	10.36	25.60	49.66	−	−
5	0.74	10.11	23.99	32.77	87.01	−
6	0.64	13.34	27.96	46.44	90.96	8.88

**Table 3:** The correlation coefficients between each VMF and the original signal.

EEG Dataset	VMF1	VMF2	VMF3	VMF4	VMF5
Freiburg	0.8598	0.5298	0.3105	0.1813	0.0773
Bonn	0.7906	0.7142	0.3614	0.1792	0.0626

**Table 4:** Epoch-based evaluation results for 21 patients in the Freiburg dataset.

Patients	Sensitivity	Specificity	Accuracy	G-Mean
1	100	99.49	99.49	99.74
2	100	96.98	96.98	98.47
3	100	97.50	97.50	98.74
4	92.35	99.03	99.01	95.63
5	100	99.89	99.89	99.94
6	100	99.08	99.08	99.53
7	100	99.83	99.83	99.91
8	100	97.01	97.01	98.49
9	98.67	96.20	96.20	97.42
10	91.52	95.95	95.49	93.70
11	100	99.96	99.96	99.97
12	100	99.98	99.98	99.99
13	100	99.85	99.85	99.92
14	98.01	98.31	98.26	98.15
15	100	98.47	98.47	99.23
16	100	98.91	98.91	99.45
17	100	99.55	99.55	99.74
18	52	96.78	96.74	70.94
19	71.21	99.97	99.97	84.37
20	100	98.79	98.79	99.39
21	95.40	98.24	97.98	96.80
Mean	95.20	98.56	98.52	96.64

**Table 5:** Event-based evaluation results for 21 patients in the Freiburg dataset.

Patients	Number of Seizures Marked by Experts	Number of Seizures Detected by Our System	Event-Based Sensitivity	False Detection Rate/h
1	2	2	100	0.35
2	1	1	100	0.70
3	3	3	100	0.89
4	4	3	75	0.15
5	3	3	100	0.03
6	2	2	100	0.20
7	2	2	100	0.07
8	1	1	100	0.29
9	3	3	100	0.80
10	3	2	67	0.87
11	2	2	100	0.06
12	3	3	100	0.09
13	1	1	100	0.07
14	3	3	100	0.43
15	2	2	100	0.12
16	3	3	100	0.26
17	4	4	100	0.10
18	3	2	67	1.41
19	2	2	100	0.03
20	3	3	100	0.16
21	4	4	100	0.43
Total	54	51	94.44	0.36

**Table 6:** Comparison of the results of our algorithm and other related works according to the Freiburg dataset.

Authors	EEG Data Duration/h	Number of Patients	Epoch-Based Sensitivity (%)	Epoch-Based Specificity (%)	Accuracy (%)	False Detection Rate/h
Yan el al. [52]	582.4	20	94.26	96.34	98.30	0.66
Mahmoodian et al. [53]	560	20	95.83	96.70	96.84	0.24
Tzimourta et al. [54]	28.6	21	99.74	−	−	0.21
Mu et al. [55]	590	21	93.2	98.16	98.16	0.5
Hussain et al. [56]	−	21	94.71	93.99	90.53	−
Abugabah et al. [57]	150	10	99.1	99	98.9	−
Malekzadeh et al. [58]	−	−	98.96	98.96	99.13	−
Our work	648.57	21	95.20	98.56	98.52	0.36

## Data Availability

Not applicable.

## Abbreviations

EEG	Electroencephalography
VMD	Variational modal decomposition
DF	Deep forest
LECM	Log-Euclidean covariance matrix
VMFs	Variational modal functions
SPD	Symmetric positive definite
MAF	Moving average filter
RF	Random forest
KNN	K-nearest neighbors
SVM	Support vector machine
BLDA	Bayesian linear discriminant analysis
CM	Covariance matrix
